# Development of the Smartphone Addiction Risk Rating Score for a Smartphone Addiction Management Application

**DOI:** 10.3389/fpubh.2020.00485

**Published:** 2020-09-11

**Authors:** Jihwan Park, Jo-Eun Jeong, Seo yeon Park, Mi Jung Rho

**Affiliations:** ^1^Department of Biomedicine and Health Sciences, College of Medicine, The Catholic University of Korea, Seoul, South Korea; ^2^Department of Psychiatry, College of Medicine, Daejeon St. Mary's Hospital, The Catholic University of Korea, Daejeon, South Korea; ^3^Computer Science and Engineering, Chung-Ang University, Seoul, South Korea; ^4^Catholic Cancer Research Institute, The Catholic University of Korea, Seoul, South Korea

**Keywords:** smartphone addiction, smartphone addiction risk rating score, Korean smartphone addiction proneness scale for adults (S-scale), nomogram, smartphone addiction management application

## Abstract

Smartphone usage characteristics are useful for identification of the risk factors for smartphone addiction. Risk rating scores can be developed based on smartphone usage characteristics. This study aimed to investigate the smartphone addiction risk rating (SARR) score using smartphone usage characteristics. We evaluated 593 smartphone users using online surveys conducted between January 2 and January 31, 2019. We identified 102 smartphone users who were addicted to smartphones and 491 normal users based on the Korean Smartphone Addiction Proneness Scale for Adults. A multivariate logistic regression analysis was used to identify significant risk factors for smartphone addiction. The SARR score was calculated using a nomogram based on the significant risk factors. Weekend average usage time, habitual smartphone behavior, addictive smartphone behavior, social usage, and process usage were the significant risk factors associated with smartphone addiction. Furthermore, we developed the SARR score based on these factors. The SARR score ranged between 0 and 221 points, with the cut-off being 116.5 points. We developed a smartphone addiction management application using the SARR score. The SARR score provided insights for the development of monitoring, prevention, and prompt intervention services for smartphone addiction.

## Introduction

Smartphones are multipurpose devices in modern life with various functions and benefits. Smartphones have diverse applications (apps) to provide user-friendly interfaces for information, communication, connection, education, and entertainment (1, 2) that promote intensive or habitual smartphone usage. Smartphones are usually used unintentionally. Unconscious smartphone users underestimate their usage time by 40% and they actually use smartphones 15% more (3). Smartphone addiction could have a negative impact on the academic performance and psychological, physical, financial, and social aspects of life (4–6). Smartphone addiction has led to uncontrolled usage of smartphones, withdrawal symptoms, and obstacles in daily life.

Smartphone addiction is an important issue regarding social behavior and mental health. It is a social problem that needs to be tackled, and various studies are underway for explore this issue. To solve this problem, identification of the risk factors that cause smartphone addiction is necessary. Therefore, previous studies have attempted to identify these risk factors and solve the negative consequences of smartphone addiction (7–9). It is important to identify the negative factors of smartphone addiction, especially based on the smartphone usage characteristics.

Smartphone usage characteristics have known risk factors. Some studies have used smartphone usage characteristics to determine smartphone addiction. Smartphone addiction is associated with smartphone usage characteristics (2, 10). Haug et al. (2) found that the duration of smartphone use on a typical day was associated with smartphone addiction in young people. Therefore, some studies developed an app to collect smartphone usage data, such as total usage duration, to assess smartphone addiction (11, 12). Lin et al. (11) proposed that non-use frequency and non-use duration significantly predicted smartphone addiction from the mobile app. Previous related apps have focused on one-dimensional usage, such as the amount of smartphone use (2, 13). Venkatesh et al. (14) found that longer duration of smartphone use, high frequency of use, shorter time until the first use of the smartphone in the morning, and social networking service (SNS) use were significantly related to smartphone addiction. Recently, studies have focused on the type and frequency of smartphone use (10, 15, 16). Some researches focused on hourly pattern and app preferences of smartphones (3).

Thus, smartphone usage characteristics are significant factors for monitoring, preventing, and intervening smartphone addiction. In terms of mental health, approaches using smartphone use characteristics are worth researching to overcome smartphone addiction, and various approaches are needed. There are many opportunities to develop behavioral intervention services for smartphone addiction. Based on these factors, development of the smartphone addiction risk rating score (SARR score) is required for the monitoring, prevention, and prompt intervention of smartphone addiction.

The smartphone addiction scale (SAS) is based on smartphone usage characteristics and the Korean self-diagnostic program for Internet addiction (K-scale) (17). The SAS consisted of the following six factors: daily-life disturbance, positive anticipation, withdrawal, cyberspace-oriented relationship, overuse, and tolerance. The SAS is an excellent scale for smartphone addiction; however, there are limitations regarding development of various intervention services by applying this scale to our app. Development of various scales for the prediction and prevention of smartphone addiction is necessary. This study indicated that the development of a risk rating score, which can predict smartphone risk by reflecting only the smartphone usage characteristics, is necessary. We required an approach to monitor and prevent smartphone addiction with the smartphones usage characteristics that we can easily collect. Here, we attempted to develop the SARR score based on smartphone usage characteristics and an app that could manage smartphone addiction using the SARR score.

## Materials and Methods

### Sample

We recruited 593 smartphone users from online surveys conducted between January 2 and January 31, 2019. All participants completed an anonymous web-based survey conducted by a polling company. The polling company delivered an email, including the online survey link and informed consent link, to the online panel. After consenting to the online survey, panel could participate in web-based questionnaires. The inclusion criteria were as follows: participants who were smartphone users and aged between 20 and 59 years. Respondents with the same answer, meaningless answer responders, and logical error responders were excluded from the study. In addition, we regarded the responses of those who responded faster than the minimum expected time as meaningless responses; therefore, we excluded them.

We identified smartphone users that were addicted to the smartphones and normal users based on the Korean Smartphone Addiction Proneness Scale for Adults (S-scale) (18). The participants' data were de-identified. Written informed consent was not required for this study in accordance with national guidelines and local legislation.

### Measures and Procedure

There were nine variables in this study: one dependent variable and eight independent variables.

First, we used the S-Scale as a dependent variable (18). The S-Scale consisted of 15 items scored with a four-point Likert scale (from 1: “not at all” to 4: “always”). The S-Scale was composed of four main categories: daily-life disturbance (five items), virtual world orientation (two items), withdrawal (four items), and tolerance (four items). The total scores were categorized into three subgroups (0–39: none; 40–43: at-risk group; and over 44: risk group). Smartphone users with S-Scale scores above 44 were evaluated as the high-risk group for smartphone addiction. The low-risk group for smartphone addiction included both a non-risk group and an at-risk group.

Second, we used the eight independent variables related to smartphone use and sleeping time (Supplementary Table 1). The smartphone use-related factors were weekday smartphone use time, weekend smartphone use time, weekly (7 days) frequency of use, process usage, social usage, habitual usage, and addictive usage.

The duration of smartphone use on a typical day is associated with the smartphone addiction in young people (2). Frequent smartphone use and frequency trends have relationships with the smartphone addiction (10, 19). Thus, we used the weekday smartphone use time, weekend smartphone use time, and weekly frequency of use as the meaningful factors in evaluating smartphone addiction.

There are two smartphone use behaviors: habitual and addictive smartphone behavior (20). We considered these two smartphone use behaviors as the smartphone use types. In this research, we perceived that smartphone addiction is a negative outcome of the smartphone use; behavior was considered as the process before the negative outcome of smartphone addiction. Thus, we focused on the habitual and addictive smartphone behavior as a risk factor for smartphone addiction.

In this study, habitual smartphone behavior meant repeated smartphone use without self-instruction or conscious thinking (20–22). The habitual smartphone behavior subscale consisted of six items scored with a five-point Likert scale (from 1: “never” to 5: “always”) (20).

Addictive smartphone behavior meant intensive smartphone use behavior (20). The addictive smartphone behavior subscale consisted of 26 items scored with a five-point Likert scale (from 1: “never” to 5: “always”) (20). In previous research, addictive smartphone behavior subscale consisted of a 10-point Likert scale. However, we used the addictive smartphone behavior subscale as a five-point Likert scale to save the respondents response time. Both the habitual and addictive smartphone behavior questionnaires measured the perception of smartphone usage patterns and characteristics.

In this study, process usage meant smartphone use for cultural and ritual processes, to receive gratification from the pleasurable experience of media content, and to realize during consumption rather than the content alone. Swanson proposed that there are two dimensions of gratifications: process and content gratification. Process gratification is derived from the pleasurable experience of media content and is realized during consumption (23, 24). Content gratifications are derived from learning information from media content and subsequently, using it in practical affairs. The process usage subscale consisted of seven items scored with a five-point Likert scale (from 1: “never” to 5: “always”) (20).

In this study, social usage meant smartphone use for social reasons. The social usage subscale consisted of five items scored with a five-point Likert scale (from 1: “never” to 5: “always”).

Finally, we used sleeping time as an independent variable. High smartphone use has a relationship with late bedtime (25). Sleeping disturbances occur across many mental health conditions (26). Sleep period markers have a relationship with the severity of depressive symptoms (27). Sleep periods can be detected by observing on/off phone screens in heavy phone users (28). Thus, we assumed that sleeping time was an important factor to be considered as an independent variable to evaluate smartphone addiction.

Based on existing literature, eight variables were selected by four smartphone addiction field professionals and experts (two psychiatrists, one medical information expert, and one data scientist). After evaluating internal consistency reliability and construct validity of four variables, we used the following items of variables: addictive smartphone behavior (*n* = 11), habitual smartphone behavior (*n* = 5), social usage (*n* = 5), and process usage (*n* = 3) (Supplementary Table 2).

### Statistical Analysis

The 593 smartphone users were analyzed using the R package (version 4_3.5.0). A multivariate logistic regression analysis was used to identify significant smartphone addiction risk factors for smartphone addiction. After the multivariate logistic regression analysis, we used the logistic regression-based nomogram to calculate the SARR score. The logistic regression-based nomogram created a risk score based on the derived logistic regression equation. The sum of each derived score was the final SARR score.

Nomogram is a visual tool that could predict the risk factors affecting the disease without complicated calculations. Even non-statistical experts can use nomograms to make decisions (29). A nomogram is created from a mathematical equation, which is typically complicated. Specially, the logistic regression-based nomogram has the benefit of providing output probability calculations based on fast and simple graphical methods. Each variable's weight is clear and expresses its relative importance (30).

We used the R Regression Modeling Strategies package (version 5.1-3) (R language; R version 3.5.0; 2018-04-23) to develop the SARR score from a logistic regression-based nomogram, using significant smartphone addiction risk factors. The logistic regression-based nomograms have long been used in diverse diseases (31, 32).

**Figure 1:**
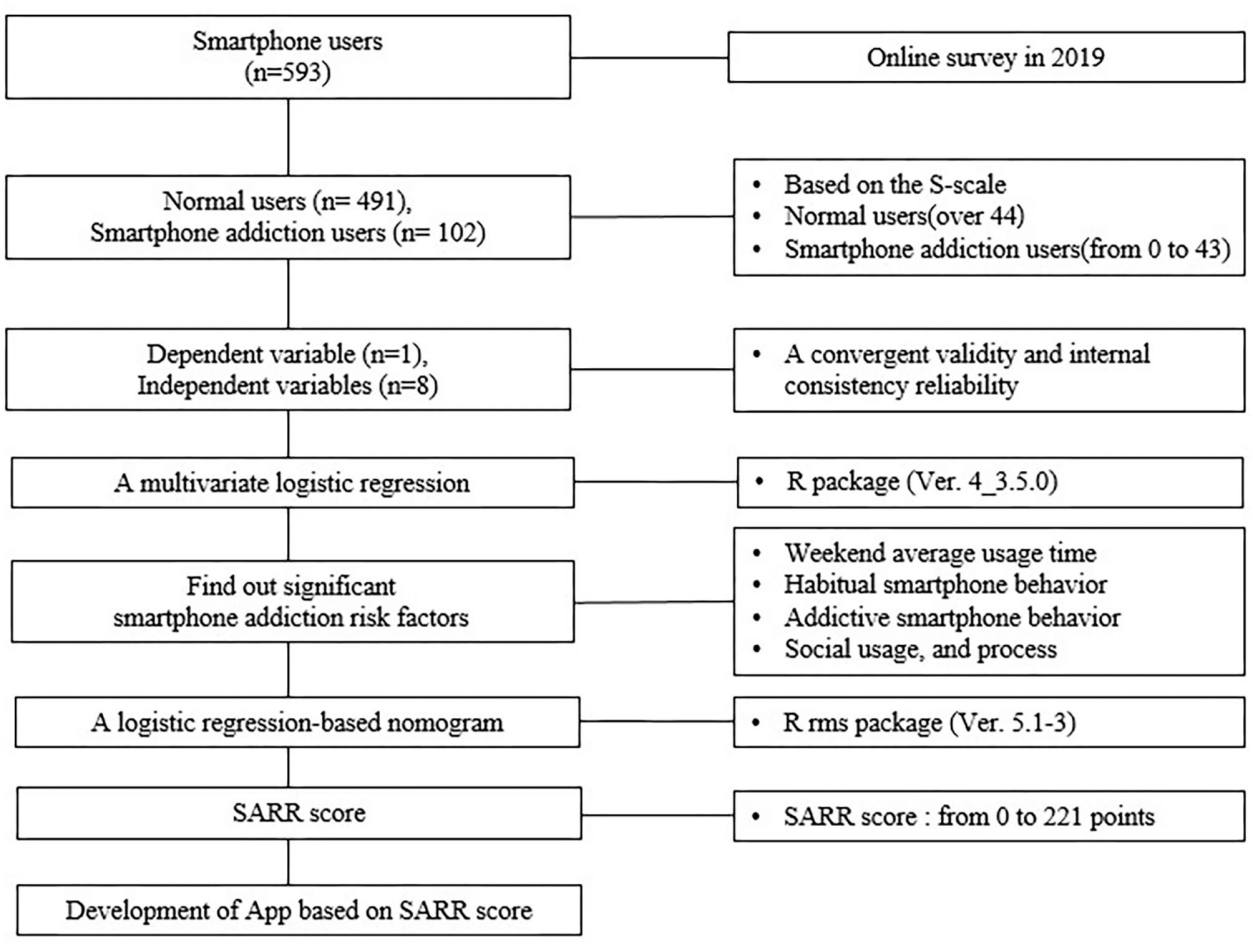
Research process.

We randomly divided the original data set into training and test data set. Data splitting is an approach for cross-validation (29). We matched six training datasets to four test datasets. Figure 1 shows the research process.

## Results

As shown in Table 1, 51.3% of the respondents were male. The respondents' ages ranged between 20 and 59 years. Of the total respondents, 55.6% were married or living with a partner. Approximately 71.3% of the respondents had occupations, such as office workers, administrative positions, service industry positions, professional technicians, freelancers, and production employees. A total of 61.9% of the respondents received over $3,584.23 as their monthly income. In addition, 66.4% of the respondents lived in the capital area, and 80.9% of the respondents had Android phones. Finally, 17.2% of the participants were in the high-risk group for smartphone addiction based on the S-scale. There were 102 users in the high-risk group and 491 users in the low-risk group.

**Table 1:** Demographic characteristics of the respondents.

**Variables**		**Frequency**	**Percentage**
Sex	Male	304	51.3
	Female	289	48.7
Age	20–29 years	132	22.3
	30–39 years	139	23.4
	40–49 years	169	28.5
	50–59 years	153	25.8
Marital status	Single^a^	263	44.4
	Couple^a^	330	55.6
Occupation	Office worker, etc.^b^	423	71.3
	Student	82	13.8
	Housewife, unemployed and other	88	14.8
Monthly income	Under $1,792.11	47	7.9
	$1,792.11–$3,584.23	179	30.2
	$3,584.23–$5,376.34	219	36.9
	Over $5,376.34	148	25.0
Residential area	Capital area (including Seoul)	394	66.4
	Non-capital area	199	33.6
Device type	Android	480	80.9
	Apple iOS	113	19.1
Group	Low-risk group for smartphone addiction	491	82.8
	High-risk group for smartphone addiction	102	17.2
Total		593	100.0

a *Single: never married, divorced, separated, or widowed; Couple: married or living with a partner*.

b *Office worker, etc.: office worker, administrative position, service industry position, professional technician, freelancer, and production employee. The exchange rate for Korean won to the US dollar is 1,116.00 won (buy and sell base rate in January 31, 2019)*.

### Risk Factors Predicting Smartphone Addiction

Four independent variables were evaluated by internal consistency reliability using Cronbach's alpha (Supplementary Table 2). The values for all constructs ranged between 0.645 and 0.928 (0.928 for addictive smartphone behavior; 0.895 for habitual smartphone behavior; 0.819 for social usage; and 0.645 for process usage). The values for three constructs were > 0.7 (33). The value of process usage was 0.645. Consequently, the Cronbach's alpha for all constructs were reliable. To test the construct validity, we performed a principal component analysis with varimax rotation. The cross-loadings were lower than the corresponding factor loadings. Four factors emerged with no-cross construct loadings above 0.50. The pattern of loadings and cross-loadings supported the discriminant validity and internal consistency. The analysis also demonstrated a convergent validity with factor loadings exceeding 0.50 for each construct. The results confirmed the existence of four factors with eigenvalues > 1.0 that accounted for 63.222% of the total variance. In addition, communality ranged between 0.496 and 0.735, with all items achieving the 0.50 threshold (Supplementary Table 2). These results confirmed that the four constructs were distinct unidimensional scales.

Table 2 shows the classification table of the multivariate logistic regression model. All variables included in the logistic regression model were free of multicollinearity. One of the measure for model performance in general linear model, Nagelkerke's *R*^2^ was 0.668. Accuracy of model was 91.4.

**Table 2:** Classification table.

	**Prediction value**			
	**Normal users**	**Smartphone users addicted to the smartphones**	**Total**	**Accuracy**
Normal users	475	16	491	96.7
Smartphone users addicted to the smartphones	35	67	102	65.7
Total	510	83	593	91.4
-2LL = 240.166, *X*^2^ = 304.272(df = 8, *p* = 0.000), Nagelkerke's *R*^2^ = 0.668				

Table 3 shows the results of the multivariate logistic regression analysis. Smartphone weekend average usage times (odds ratio [OR] =1.002) were significant predictors of smartphone addiction. In addition, the process usage, social usage, habitable usage, and addictive usage of smartphones were significant factors that predicted the smartphone addiction (OR =1.160, 0.786, 1.267, and 1.162, respectively).

**Table 3:** Risk factors predicting smartphone addiction.

**Parameter**	**Estimate**	**SE**	***p*-value**	**OR 95% CI**
Intercept	−17.137	2.215	0.000	
Smartphone use weekday time	−0.001	0.001	0.449	0.999 (0.997–1.001)
Smartphone weekend averages usage time	0.002	0.001	0.040^**^	1.002 (1.000–1.004)
Weekly frequency to use	0.000	0.001	0.635	1.000 (0.998–1.001)
Sleeping time	−0.003	0.002	0.126	0.997 (0.993–1.001)
Process usage	0.149	0.057	0.009^**^	1.160 (1.038–1.297)
Social usage	−0.241	0.063	0.000^***^	0.786 (0.695–0.889)
Habitual smartphone behavior	0.236	0.060	0.000^***^	1.267 (1.126–1.425)
Addictive smartphone behavior	0.150	0.017	0.000^***^	1.162 (1.123–1.203)

*SE, standard error; OR, odds ratio; CI, confidence interval*.

** *p < 0.01*;

*** *p < 0.001*;

*Time unit: minute*.

### The SARR Score Based on the Logistic Regression-Based Nomogram

We developed the SARR score based on the logistic regression-based nomogram as depicted in Figure 1. The total prediction rate was 91%, which was considered reasonably accurate. The specificity was 96% and the sensitivity was 66%. Thus, this study accepted these results.

**Figure 2:**
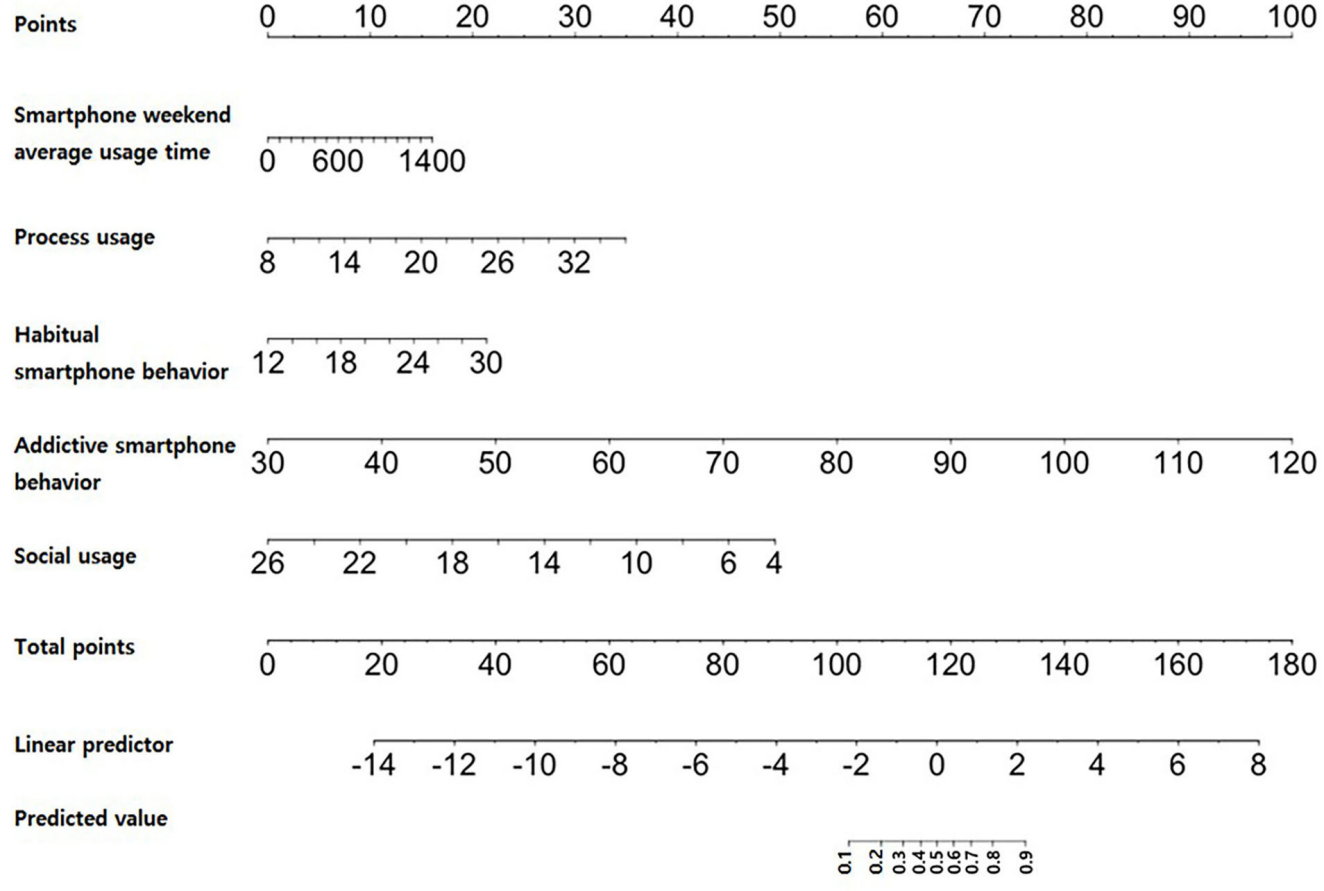
Nomogram of the SARR score.

Figure 2 shows the nomogram of SARR score. The SARR score nomogram consisted of a series of measures corresponding to each variable in the equation. It could freeze the values of point variables to check the relationship between the following non-fixed variables: smartphone weekend average usage time, process usage, habitual smartphone behavior, addictive smartphone behavior, and social usage. We could obtain point values of a variable by placing a straight line against a point value of the scale, a value at a location that intersects the scale of that variable. A predicted value intersects the total points, which is summation of point values of these variables.

The total risk rating score was calculated as the sum of the values for each variable (Table 4). The SARR score ranged between 0 and 221 points. The cutoff of the SARR score was 116.5 points. In the receiver operating characteristic curve, the cutoff value represented the portion where the sensitivity and specificity of the graphs overlapped (sensitivity: 0.823 and specificity: 0.831) (Supplementary Figures 1, 2).

**Table 4:** Smartphone addiction risk rating score.

**Variable**		**RRC**	**Variable**		**RRC**	**Variable**		**RRC**	**Variable**		**RRC**	**Variable**		**RRC**
Smart phone weekend average usage time	0	0	Process usage	8	0	Social usage	4	49	Habitual smartphone behavior	12	0	Addictive smartphone behavior	30	0
	100	1		10	2		6	45		14	2		40	11
	200	2		12	5		8	40		16	5		50	22
	300	3		14	7		10	36		18	7		60	33
	400	5		16	10		12	31		20	9		70	44
	500	6		18	12		14	27		22	12		80	56
	600	7		20	15		16	22		24	14		90	67
	700	8		22	17		18	18		26	17		100	78
	800	9		24	20		20	13		28	19		110	89
	900	10		26	22		22	9		30	21		120	100
	1000	11		28	25		24	4						
	1100	13		30	27		26	0						
	1200	14		32	30									
	1300	15		34	32									
	1400	16		36	35									

*Time unit, minute; RRC, risk rating score*.

**Figure 3:**
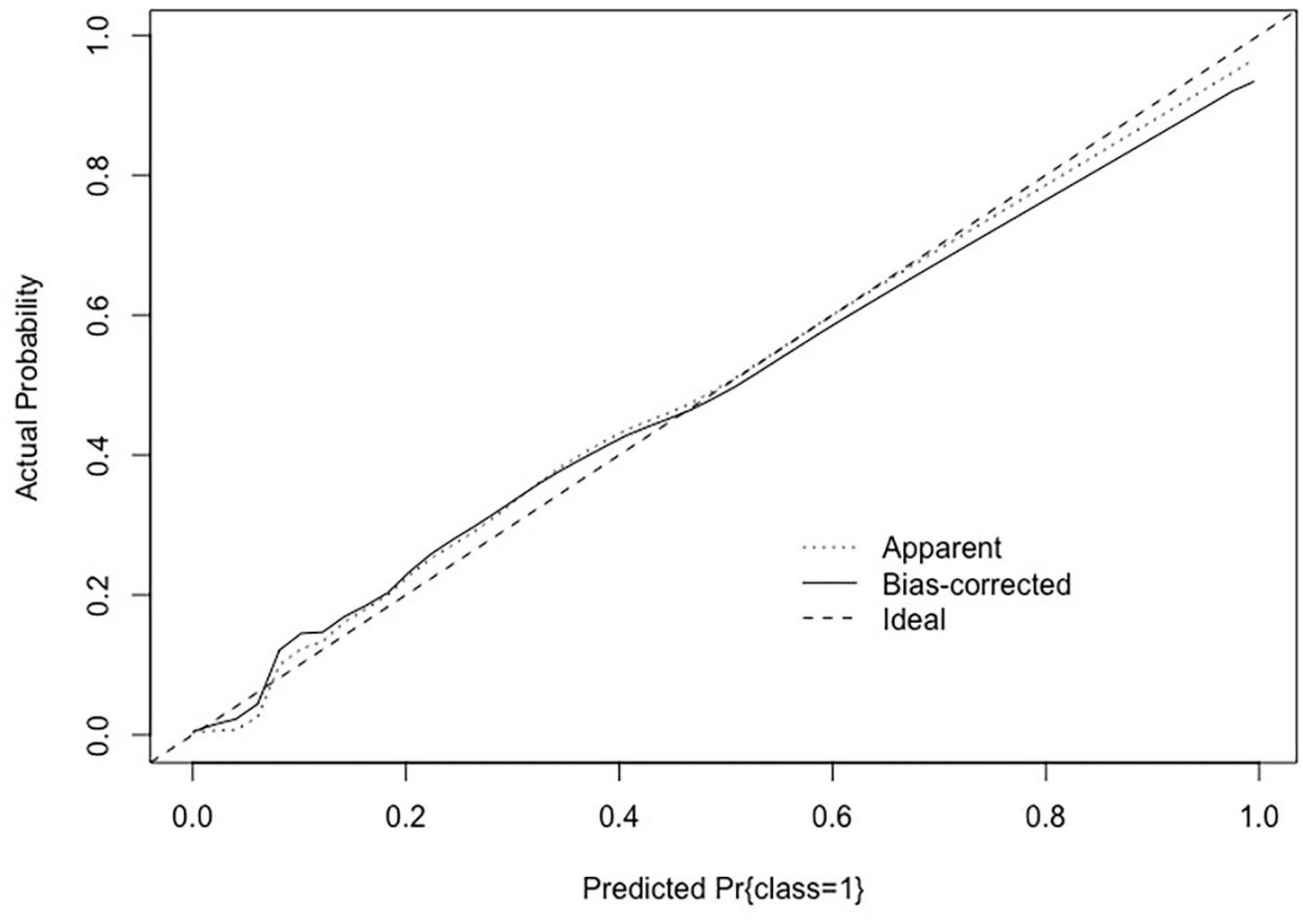
Calibration of the SARR score.

Figure 3 shows the calibration of SARR score. The calibration was assessed by categorizing the smartphone users by their SARR score. The calibration was assessed based on the plot of predicted probabilities between the nomogram and actual probabilities (29). The calibration of smartphone addiction illustrated how its predictions were compared with the actual outcomes of the 593 smartphone users (34). The x-axis is the predicted value calculated using the SARR score, and the y-axis is the actual smartphone addiction probability of the smartphone users (35). The best predictions corresponded to the 45° line. Points estimated below the 45° line represented over-prediction, and those above the 45° line represented under-prediction (36). The SARR score prediction line was close to 45° and the results were appropriate.

### Smartphone Addiction Management App Based on the SARR Score

**Figure 4:**
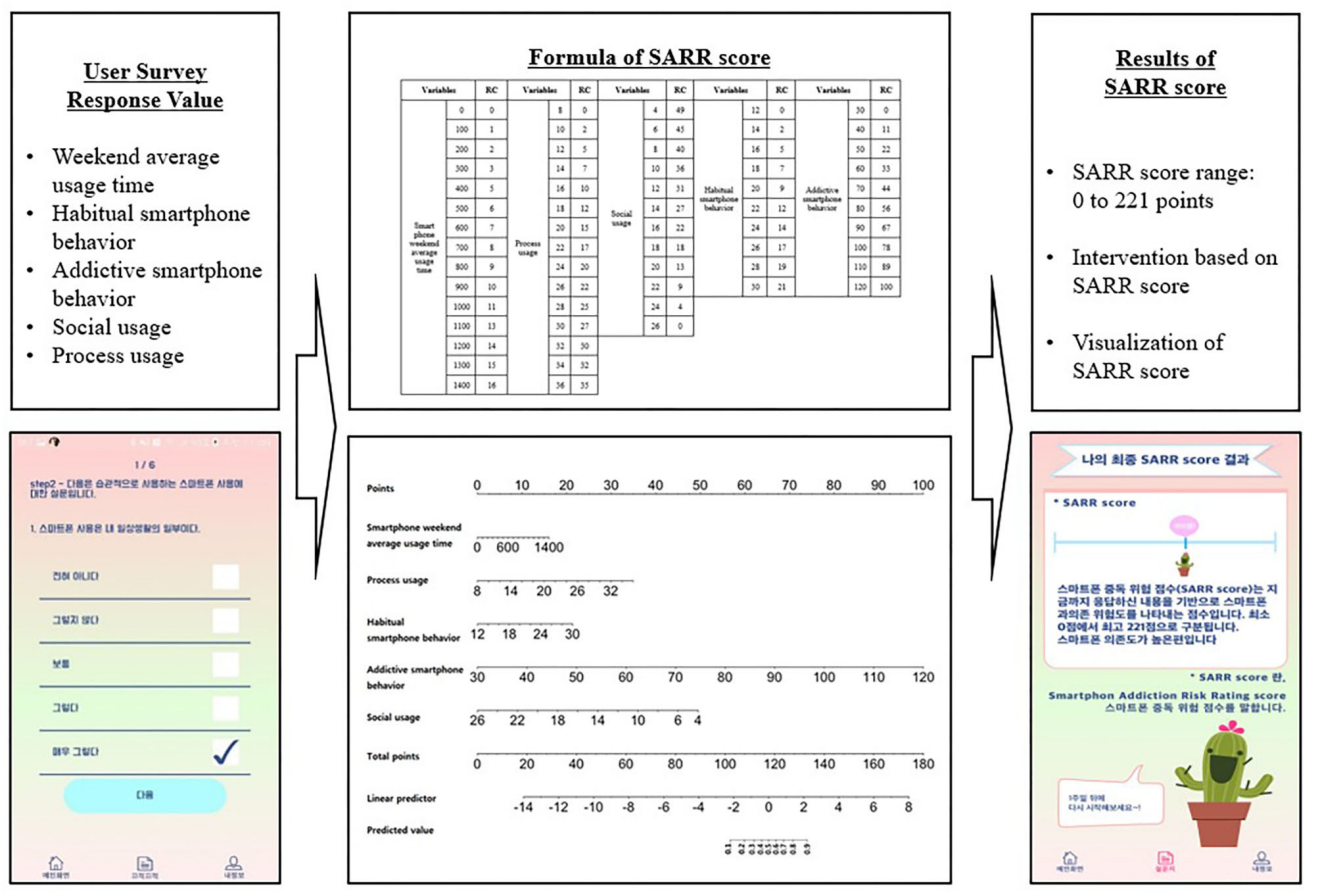
App process based on the SARR score.

We developed the smartphone addiction management app to manage smartphone addiction using the SARR score. Figure 4 shows the app process based on the SARR score. Survey response values of a user obtained from questionnaires through the app related to weekend average usage time, habitual smartphone behavior, addictive smartphone behavior, social usage, and process usage are presented in Figure 4. These response values are inputs of the formula of the SARR score as presented in the center of Figure 4. This formula calculates the SARR score using the user's questionnaire response values as the input values. The SARR score was visualized on the result screen of the app. An intervention service can be provided to manage the smartphone addiction according to the derived SARR score.

## Discussion

We identified five relevant factors among smartphone usage characteristics and developed the SARR score based on meaningful factors. Based on the results of this study, the following conclusions were derived:

First, smartphone weekend average usage time, process usage, social usage, habitable usage, and addictive usage of smartphones were relevant variables in smartphone addiction. In particular, smartphone weekend average usage time was the only important factor in terms of smartphone addiction among these factors.

The number of hours spent on smartphone use explains the smartphone addiction (37). However, weekly frequency of use and weekday smartphone use time had no relationship with smartphone addiction. Most people keep their smartphones on, use them frequently, and rely on them. On average, people check their smartphone 46 times per day (38). Thus, normal smartphone users also use smartphones frequently on the weekend and have a high frequency of smartphone use. Frequent smartphone use is associated with smartphone addiction (10, 11, 19). Our results differ from those of previous studies regarding frequent smartphone use been associated with smartphone addiction. Recently, studies have focused on the type of smartphone use and frequency of smartphone use (15). Thus, a separate approach is needed for studying smartphone use time for smartphone addiction in detail with regard to the following factors: smartphone weekday usage time and weekend usage time.

We also found that process usage and social usage influenced smartphone addiction. Currently, many people have relationships with other people through their smartphones and the cyber world (39). Social networking is a personal and relevant smartphone function and has an association with smartphone addiction (2). The SNS use was a stronger predictor of the smartphone addiction than the game use (40). Social reasons also lead to the highest levels of addiction to the Internet (41). Smartphones have diverse functions of providing a user-friendly interface for information, communication, education, and entertainment. Smartphone users receive gratification from the pleasurable experience of smartphone content, and the gratification is realized during consumption. Smartphones are specialized in using mobile messengers and SNS, it can be used for social interaction and relationships anytime, anywhere (42). This smartphone usage characteristic may lead to smartphone addiction.

In addition, we focused on two concepts of smartphone usage characteristics: habitual and addictive smartphone behavior. This study also found that habitual and addictive smartphone behaviors make smartphone use more pervasive. Oulasvirta et al. proposed that habits make smartphone use more pervasive (22). Habitual behavior is an automatic response to internal and external cues (43). Van Deursen et al. also proposed the concept of habitual and addictive smartphone behavior (20). Habitual smartphone behavior influences addictive smartphone behavior and smartphone addiction. However, habitual smartphone behavior is not addictive smartphone behavior and smartphone addiction. Accordingly, separate concepts of smartphone use are needed.

Second, we developed the SARR score based on five relevant factors as follows: smartphone weekend average usage time, process usage, social usage, habitable usage, and addictive usage. The SARR score ranged between 0 and 221 points. If we collected the data for these five factors, we could express the risk score visually in intervention service. The risk score could be used to develop smartphone addiction monitoring, prevention, and prompt intervention. We are developing smartphone addiction monitoring, prevention, and prompt intervention services using the SARR score. We hope that these services will help smartphone users to control their smartphone use by themselves.

Third, we found that sleeping time was not an important factor for smartphone addiction. Previous researchers have proposed that feeling good is greatly influenced by receiving enough sleep (44). Sleep plays a significant role in mental health. Many studies have focused on sleep as a behavioral marker in mental health and smartphone addiction (27, 45–48). We found no relationship between sleeping time and smartphone addiction. A previous study reported that the sleep duration on weekends and midpoint of sleep on weekdays and weekends did not predict the smartphone addiction (25). In addition, previous researches proposed that sleep quality mediated the relationship between problematic smartphone use and health symptoms, such as body dysfunction (49). Thus, sleep time can be a significant variable in intervention of smartphone addiction, but more research is needed.

Fourth, we focused on a logistic regression-based nomogram to develop the risk score in smartphone addiction. As explained earlier, the logistic regression-based nomogram has the benefit of providing output probability calculations based on fast and simple graphical methods. In addition, each variable's weight is clear and expresses its relative importance (30). Although the logistic regression-based nomograms have long been used in diverse diseases (31, 32, 50, 51), fewer attempts have been made to use nomograms in mental health studies. Our attempt represents a meaningful approach.

Finally, although there is the SAS based on the K-scale (17), we still developed the SARR score. The SAS consisted of the following six factors: daily-life disturbance, positive anticipation, withdrawal, cyberspace-oriented relationship, overuse, and tolerance. The SAS has good psychometric qualities. The SAS is a suitable scale for judging smartphone addiction; however, there are limitations regarding development of various intervention services by applying this scale to the apps. This is because SAS does not specifically enquire regarding smartphone usage characteristics. Thus, we developed the SARR score to further develop a smartphone addiction management app. We had to evaluate smartphone addiction risk status of the users periodically for app service. It is not reasonable to ask psychological questions to smartphone users every time for the intervention of smartphone addiction. App users are cumbersome and have a psychological burden. We judged that it is necessary to develop the SARR score that can predict smartphone risk rate by reflecting only the smartphones usage characteristics, which can be collected easily. The SARR score will be useful for all services through smartphone intervention apps.

Although significant results were obtained from this study, it had some limitations. First, we used smartphone use data from a self-reported questionnaire. Future research should assess real smartphone use by collecting data from mobile devices. Second, we only focused on the smartphone usage characteristics. Future research should focus on other psychiatric symptoms, such as depression and anxiety (4). Third, there is no previous related risk score based on only smartphone usage characteristics for smartphone addiction. Hence, we could not compare our results with other risk models. Fourth, although there are variables related to smartphone use, various literatures do not exist yet. A lot of in-depth research is needed. Fifth, we used the term “smartphone addiction” in this study. Various related terms, such as problematic smartphone use, excessive smartphone use, smartphone dependence, and smartphone addiction, are used (52–55). There is no consensus within the academic community or representation in clinical manuals that a particular term is recognized. We recognized the controversy of this issue. Hence, if agreement on terms is reached; it would be desirable to interpret this study in terms of the term agreed upon in the future. Sixth, in the case of sleep time, various measurements are possible, such as App sensing data or Pittsburgh sleep quality index (56). In this study, sleep time was measured only by one item in the self-reported questionnaire; hence, it is necessary to collect and analyze the sleep time by other methods. Seventh, the SARR score is intended to monitor the smartphone addiction risk rate and status, rather than be a tool for judging smartphone addiction. In the future, for the psychiatrists to use it in determining smartphone addiction, further research will be needed based on actual smartphone usage. Finally, we used the S-Scale scored with a four-point Likert scale to identify 102 smartphone users that were addicted to the smartphones and 491 normal users. In case of a four-point Likert scale, there was no medium point in the measurement of the items. Using a four-point scale is problematic and could have potentially distorted the results. However, to use the S-Scale cut-off, the five-point scale of the original S-Scale must be used. When the scale is adjusted to five points, the cutoff is arbitrarily changed. Future research should use the five-point S-Scale to identify the smartphone addiction group with a new cutoff value. This can be evaluated as a different topic from this study.

## Conclusions

This study developed the SARR score that can predict smartphone risk by reflecting only the smartphone usage characteristics. It was developed for smartphone addiction monitoring, prevention, and providing prompt and timely intervention services. We further developed the smartphone addiction management app using the SARR score. The developed app may be efficiently used for managing actual smartphone use. In addition, the SARR score could be extended to other mental health issues and contexts.

## Data Availability Statement

The raw data supporting the conclusions of this article will be made available by the authors, without undue reservation.

## Ethics Statement

The studies involving human participants were reviewed and approved by Institutional Review Board of Catholic University (IRB number: MC18QNSI0101). Written informed consent for participation was not required for this study in accordance with the national legislation and the institutional requirements.

## Author Contributions

The study was designed by JP and MR. Data collection was performed by MR. Statistical analyses have been conducted by JP and MR, whereas J-EJ advised on some of the clinical approaches. The manuscript has been written by MR. In detail, JP drafted the complete method and statistical analysis section, whereas J-EJ and MR together drafted the introduction. JP, J-EJ, and MR drafted the discussion, which was then clinically revised by J-EJ. JP, J-EJ, and MR independently checked all statistical results. SP developed the app to manage smartphone addiction. The final version has been approved by all authors.

## Conflict of Interest

The authors declare that the research was conducted in the absence of any commercial or financial relationships that could be construed as a potential conflict of interest.
