# Supplementary material for: Development of the Smartphone Addiction Risk Rating Score for a Smartphone Addiction Management Application
Source: Front Public Health. 2020 Sep 11;8:485. doi: 10.3389/fpubh.2020.00485 (PMC7517726; doi:10.3389/fpubh.2020.00485)

**Supplementary** **Table1** Questionnaire (English version).

| **No** | **Independent variables and Questionnaire** |
| --- | --- |
| **1** | **Smartphone usage weekday time**  How many hours a day have you used your smartphone on average over the past week?  ▶ Weekdays (average __ hours __ minutes) |
| **2** | **Smartphone weekend averages usage time**  How many hours a day have you used your smartphone on average over the past week?  ▶ Weekend (average __ hours __ minutes) |
| **3** | **Weekly frequency to use**  How many times have you used your smartphone in the past week? ( ) times |
| **4** | **Sleeping time**  In the past month, how many hours per day have you been sleeping on average? please write down the time you actually slept, not the time you lie down. (average __ hours __ minutes) |
| **5** | **Process usage (5-point scale)**  1. I use my smartphone in order to escape from real-life.  2. I use my smartphone in order to relax.  3. I use my smartphone because it is entertaining.  4. I use my smartphone because it informs me for things that happen in everyday life.  5. I use my smartphone in order to stay up to date of the latest news.  6. I use my smartphone because it helps me passing time.  7. I use my smartphone because it’s a pleasant break from my routines. |
| **6** | **Social usage (5-point scale)**  1. I use my smartphone to interact with people.  2. I use my smartphone to maintain relationships.  3. I use my smartphone to call other people.  4. I use my smartphone to text message others.  5. I use my smartphone to contact people through social media. |
| **7** | **Habitual smartphone behavior (5-point scale)**  1. Smartphone usage is part of my daily routines.  2. Checking my smartphone is becoming a habit.  3. I use my smartphone automatically.  4. It’s a habit to use my smartphone.  5. My smartphone is a part of my life.  6. When I need to complete a certain task than the use of my smartphone is an obvious choice. |
| **8** | **Addictive smartphone behavior (5-point scale)**  1. I can never spend enough time on my mobile phone. ‘  2. I have used my mobile phone to make myself feel better when I was feeling down.  3. I experience problems when I find myself using my mobile phone when I should be doing other things.  4. I have tried to hide from others how much time I spend on my mobile phone.  5. I lose sleep due to the time I spend on my mobile phone.  6. I have spent with the mobile phone more than I should have.  7. When out of range for some time, I become worried about the thought of missing a call.  8. Sometimes, when I am on my mobile phone and I am doing other things, I get carried away with the conversation and I don’t pay attention to what I am doing.  9. The time I spend on my mobile phone has increased over the last 12 months.  10. I have used my mobile phone to talk to others when I was feeling isolated.  11. I have attempted to spend less time on my mobile phone but am unable to.  12. I find it difficult to switch off/switch to silent my mobile phone.  13. I feel anxious if I have not checked for messages or switched on my mobile phone for some time.  14. I have frequent dreams about my mobile phone.  15. My friends and family complain about my use of the mobile phone.  16. If I don’t have a mobile phone, my friends would find it hard to get in touch with me.  17. My academic performance has decreased as a direct result of the time I spend on my mobile phone.  18. I have aches and pains that are associated with my mobile phone use.  19. I find myself using on my mobile phone for longer periods of time than intended.  20. There are times when I would rather use my mobile phone than deal with other more urgent matters.  21. I am often late for appointments because I’m talking on my mobile phone when I shouldn’t be.  22. I become irritable if I have to switch off/to silent my mobile phone for classes, meals, or at the cinema.  23. I have been told that I spend too much time on my mobile phone.  24. More than once I have been in trouble because my mobile phone has gone off during a class, at the cinema, or in a restaurant.  25. My friends don’t like it when my mobile phone is switched off/to silent.  26. I feel lost without my mobile phone. |

**Supplementary** **Table2** Loadings, cross-loadings, and reliability.

| **Variables** | | **1** | **2** | **3** | **4** | **Communality** | **Cronbach’s α** |
| --- | --- | --- | --- | --- | --- | --- | --- |
| **Addictive smartphone behavior** | ASB17 | **.822** | .118 | -.017 | .007 | ` | 0.928 |
|  | ASB22 | **.809** | -.082 | .020 | .122 | .676 |  |
|  | ASB23 | **.807** | .182 | -.057 | .013 | .688 |  |
|  | ASB15 | **.788** | .088 | -.015 | -.011 | .629 |  |
|  | ASB14 | **.779** | -.145 | .040 | .063 | .633 |  |
|  | ASB21 | **.777** | -.011 | .052 | .021 | .608 |  |
|  | ASB20 | **.743** | .319 | .023 | -.082 | .662 |  |
|  | ASB24 | **.733** | .134 | .049 | .073 | .563 |  |
|  | ASB18 | **.710** | .121 | -.053 | -.052 | .524 |  |
|  | ASB08 | **.653** | .353 | -.057 | -.074 | .559 |  |
|  | ASB11 | **.647** | .366 | .026 | -.039 | .555 |  |
| **Habitual smartphone behavior** | HSB04 | .163 | **.830** | .138 | .031 | .735 | 0.895 |
|  | HSB02 | .043 | **.820** | .189 | .090 | .718 |  |
|  | HSB01 | .132 | **.794** | .185 | .094 | .691 |  |
|  | HSB05 | .149 | **.787** | .166 | .120 | .684 |  |
|  | HSB03 | .222 | **.775** | .102 | .061 | .665 |  |
| **Social usage** | SU02 | .120 | .127 | **.795** | .178 | .695 | 0.819 |
|  | SU01 | .058 | .164 | **.771** | .148 | .647 |  |
|  | SU04 | -.144 | .131 | **.745** | .047 | .595 |  |
|  | SU03 | -.206 | .168 | **.726** | .033 | .599 |  |
|  | SU05 | .177 | .098 | **.670** | .144 | .511 |  |
| **Process usage** | PU06 | .024 | .099 | .171 | **.817** | .708 | 0.645 |
|  | PU04 | .069 | .054 | .145 | **.785** | .645 |  |
|  | PU05 | -.142 | .356 | .311 | **.502** | .496 |  |
| **Eigenvalue** | | 6.507 | 3.925 | 3.049 | 1.692 |  | |
| **% of Variance** | | 27.113 | 16.355 | 12.705 | 7.049 |  |  |
| **Cumulative %** | | 27.113 | 43.468 | 56.173 | 63.222 |  |  |
| **Number of questions** | | 11 | 5 | 5 | 3 |  | |

**ASB: Addictive smartphone behavior, HSB: Habitual smartphone behavior, SU: Social usage, PU: Process usage, Extraction Method: Principal Component Analysis, Rotation Method: Varimax with Kaiser normalization.*

**Supplementary** **Figure1** AUC Curve.


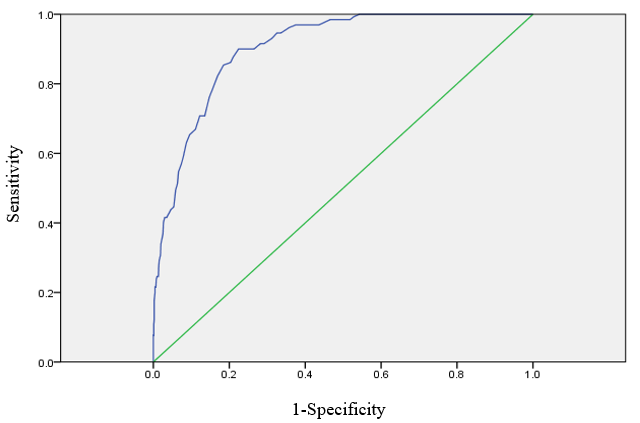


**Supplementary** **Figure2** Cutoff value of the SARRS**.**


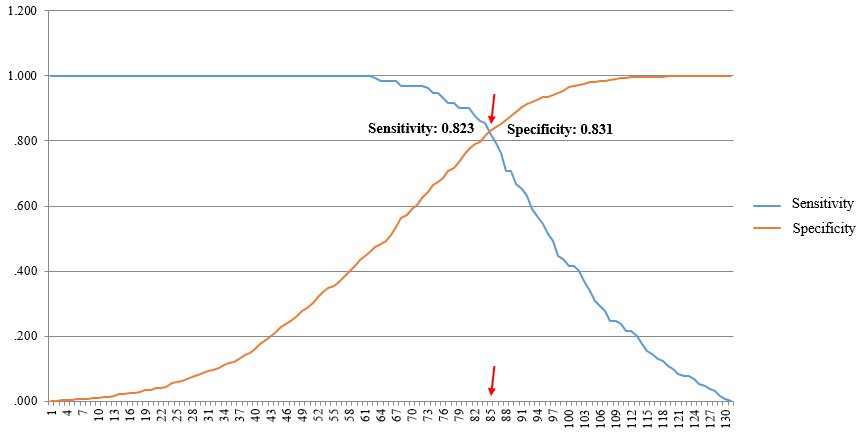

Supplement: Supplementary file 1 [file Data_Sheet_1.docx]
